# A peculiarly characterised case of isoniazid-induced pellagra- 2 Ds and a C: a case report

**DOI:** 10.11604/pamj.2021.39.73.28072

**Published:** 2021-05-26

**Authors:** Chishiba Kabengele, Hellen M’hango, Diana Mweemba, Malan Malumani

**Affiliations:** 1 Research Physician-Rwanda Zambia Health Research Group, Lusaka, Zambia,; 2 Research Fellow-Sub-Saharan Network for TB and HIV Research Excellence (SANTHE), Durban, South Africa,; 3 Medical Residency, Mazabuka General Hospital, Mazabuka, Zambia,; 4 Registrar, Children´s Hospital- The University Teaching Hospitals, Lusaka, Zambia,; 5 Registered Nurse, Mazabuka General Hospital, Mazabuka, Zambia,; 6 Dermatology and Venereology Unit, Department of Internal Medicine, Livingstone Central Hospital, Livingstone, Zambia,; 7 Mulungushi University, School of Medicine and Health Sciences, Kabwe, Central Province, Zambia

**Keywords:** Pellagra, niacin deficiency, isoniazid-induced pellagra, photosensitive dermatitis, case report

## Abstract

Niacin or tryptophan deficiency causes pellagra. Isoniazid interferes with the absorption of niacin and individuals on Isoniazid (INH) are at risk of pellagra. Isoniazid preventive therapy (IPT) is the administration of isoniazid to immunosuppressed individuals to prevent active tuberculosis (TB). IPT, in sub-Saharan Africa, the region worst hit by HIV and with a high TB prevalence, is recommended. A 40-year-old, HIV+ Zambian woman on Antiretroviral therapy for five years and IPT for three months presented with a four-day history of constipation, generalised body weakness and irrelevant talk. She complained of a generalised rash, sloughing off, and darkening of the skin on the face, neck, forearms, and dorsum of both feet. A physical examination revealed features of pellagra, and rapid response to oral niacin reaffirmed the diagnosis of pellagra. Unlike typical cases of pellagra presenting with the classic 3 Ds of Diarrhoea, Dementia and Dermatitis, our patient presented with constipation instead of diarrhoea. A consideration of Pellagra in HIV+ patients on IPT whose diet is mostly maize-based will be beneficial, even if the classic 3 Ds of diarrhoea, dementia, and dermatitis are not wholly present. A timely diagnosis and prompt treatment of pellagra can be lifesaving.

## Introduction

Gasper Casal first described pellagra in 1735 in Spain [1]. It is the archetypal clinical exhibition of niacin deficiency or its precursor amino acid tryptophan and is depicted by erythema and hyperpigmentation of the skin in sun-exposed areas. Niacin (vitamin B3) is a combination of two compounds: nicotinamide and nicotinic acid [2]. Niacin plays a significant role in the metabolism of alcohol, fats, carbohydrates, and proteins. It is also essential in cell signalling, nucleic acid repair, and the detoxification of drugs and reactive oxygen species [3]. The systemic disease that results from niacin deficiency clinically manifests as the 4 Ds: photosensitive dermatitis, dementia, diarrhoea, and death in severe cases [4].

Globally, tuberculosis (TB) is the leading cause of death from a single infectious agent and accounted for 1.4 million deaths in 2019. Human Immunodeficiency Virus (HIV) infection is the most substantial risk factor for tuberculosis (TB) disease and people living with HIV (PLHIV) are eighteen (18) times more likely to develop active TB than those that do not have HIV [5]. To prevent the evolution of latent tuberculosis to active disease, isoniazid (INH) preventive therapy (IPT) is commonly indicated in PLHIV and other immunosuppressed individuals. The duration of IPT is usually six months. Despite being classically associated with distorted pyridoxine metabolism, INH also indirectly affects tryptophan conversion to nicotinamide. Pellagra may, therefore, result from INH despite pyridoxine supplementation [6].

HIV infection provokes a pellagra-like condition; plasma tryptophan levels are reduced in patients with HIV infection, and elevated dosages of nicotinamide treatment could effectively reverse this HIV-associated metabolic irregularity [7]. HIV infection may induce niacin depletion. Adding INH preventive therapy may worsen the deficit of plasma niacin levels, especially in the malnourished and in those whose staple food is maize-based. We report a case of isoniazid-induced pellagra in an HIV positive patient on isoniazid prophylaxis in Zambia with constipation rather than the expected and traditional diarrhoea.

## Patient and observation

**Patient information:** a 40-year-old female presented to the outpatient department with a four-day history of constipation, generalised body weakness, irrelevant talk, and inability to walk. She was unable to feed orally for two days before presenting to the hospital. She also complained of a generalised rash, sloughing off, and darkening of the skin on the face, neck, forearms, and dorsum of both feet. The rash was itchy and started on the forehead and spread to the cheeks and chin. The patient is HIV positive and has been on antiretroviral therapy for five (5) years now (lamivudine, tenofovir, and efavirenz). She was also on Isoniazid prophylactic therapy (300 mg) and pyridoxine (50 mg) for three months at the time of presentation. She was treated for bacteriologically confirmed TB 5 years before she presented with the above symptoms. She had quit alcohol when she was diagnosed with TB. Her main diet was corn-based, with little protein and occasional vegetables, and she lived with her two children and her cousins and was widowed.

**Figure 1:**
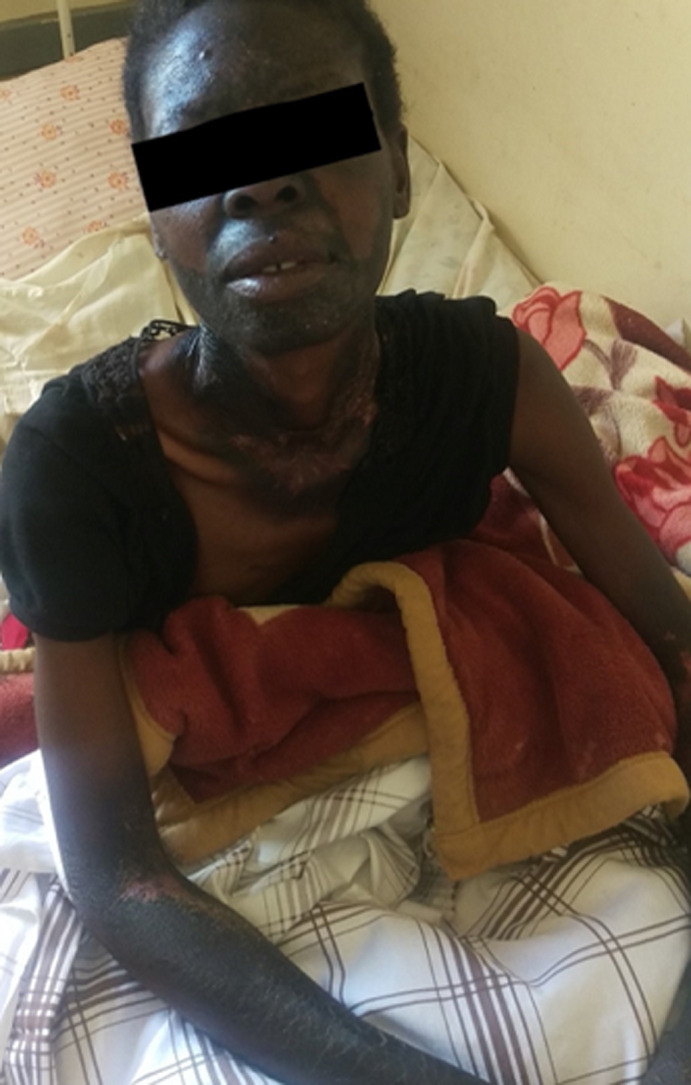
pre-treatment appearance of the patient showing photodermatitis, hyperpigmentation in sun-exposed areas and casal’s necklace (courtesy of Dr Chishiba Kabengele)

**Clinical findings:** when examined, she was weak and had vital signs within normal limits: blood pressure 110/61mmHg; temperature 36.5°C; pulse 98/min; respiratory rate 18/min. Her speech was slurred. Her tongue was smooth on the oral cavity examination. She appeared distressed and had hyperpigmented lesions with fissures on the forehead, nose, zygomatic and perizygomatic regions, around the neck (Casal's necklace), on both forearms, on the dorsal areas of both feet, as seen in Figure 1. Digital Rectal Examination revealed hard, impacted stool. The rectal mucosa was smooth, and no other masses were noted. Examination of the other systems was unremarkable.

**Diagnostic assessment:** laboratory tests that were done are shown in Table 1. She had microcytic, normochromic anaemia, elevated ESR and HIV-1 viral load and a low CD4 count. The rest of the parameters were within reference ranges. A clinical diagnosis of isoniazid-induced pellagra in an HIV patient with constipation was entertained based on the appearance, distribution, and time frame over which the rash developed.

**Table 1:** results of laboratory investigations that were done

Laboratory test	Results	Reference range
White blood cell (*109/L)	3.32	3.5-10.0
Lymphocytes (*109/L)	0.9	0.9-5.0
Granulocytes (*109/L)	2.25	1.2-8.0
Hemoglobin (g/dl)	10	11.5-16.5
Mean cell hemoglobin (pg)	26.7	25.0-35.0
Mean corpuscular volume (fl)	73.9	75.0-100.0
Platelets (*109/L)	286	150-450
Erythrocyte sedimentation rate (ESR)	18	< 15
Hepatitis B surface antigen	Negative	
Viral load	250 copies/ml	< 50 copies
Treponema pallidum test (RPR)	Negative	
CD 4 Count (cells/UI)	368	400-1200
AST (U/L)	20.4	8.0-45.0
ALT (U/L)	23.3	8.0-45.0
Creatinine (mmol/L)	32	60-120
Urea	2.4	2.0-7.0

**Therapeutic intervention:** the patient was hospitalised, and the management plan initially included intravenous vitamin B complex (2mls OD), rectal evacuation with a soap enema, and lactulose syrup. Isoniazid was temporarily withdrawn and reintroduced later after dietary counselling and niacin supplements. Vitamin B3 was introduced on the second day of admission. After dermatological consultation, the entertained diagnosis of pellagra was maintained, and niacin capsules for eight weeks were prescribed. Due to the high cost of niacin, vitamin B complex tablets were instead used by the patient, and Pyridoxine dose was adjusted upwards to 50 mg twice a day for 12 weeks.

**Figure 2:**
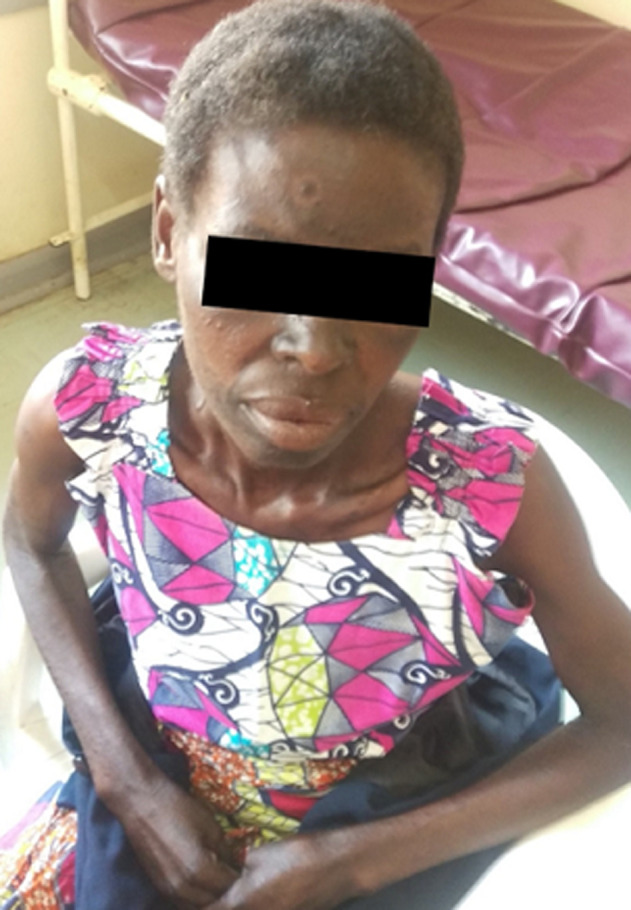
two weeks post-treatment, showing resolving lesions (courtesy of Dr Chishiba Kabengele)

**Follow-up and outcomes:** the patient opened their bowels, on her own, after 48 hours of treatment with lactulose. After two weeks, the patient was reviewed, there was marked improvement of the rash, as shown in Figure 2. Her bowel habits had returned to normal after discharge from the hospital. The patient continued a high protein diet of High energy protein supplement (HEPS) and animal protein, and the generalised body weakness had resolved after a week of treatment. She was scheduled to return for review after a month but did not return.

**Patient perspective:** during the time she was hospitalized and after the treatment, the patient was delighted with the care she received and was optimistic about the outcome of her condition.

**Informed consent:** the patient was informed about the case report, why her case was peculiar and the authors’ interest in publishing her case. She willingly gave informed consent to allow the authors to use her photos for this case report.

**Patient´s consent:** informed consent was obtained from the client for us to use the pictures.

## Discussion

Pellagra can be primary or secondary [8]. It is termed primary pellagra when it is a result of deficiency of niacin or vitamin B3. If it occurs, when dietary intake of niacin is adequate, as a result of conditions that affect tryptophan or niacin metabolisms such as alcoholism and use of drugs such as isoniazid, it is termed as secondary. Isoniazid is structurally similar to niacin and therefore competes, biochemically, with niacin resulting in inhibition of niacin's absorption at the intestinal level. The competitive absorption of niacin and isoniazid results in the accumulation of isoniazid, which then inhibits niacin's endogenous production, leading to pellagra [8]. In our patient's case, we contemplate that isoniazid, coupled with her poor diet and HIV infection, was the direct cause of pellagra.

Niacin is an essential component in coenzyme I and coenzyme II, which either donate or accept hydrogen ions in vital oxidation-reduction reactions and, therefore, is needed for adequate cellular function and metabolism. Nicotinamide, the amide form of niacin to which it is converted in the body, on the other hand, has immunomodulatory and anti-inflammatory effects. These essential cellular functions and roles that niacin performs in multiple organs and tissues lead to a diversity in the symptoms and signs associated with pellagra.

The gastrointestinal disturbances that arise include epigastric discomfort, reduced appetite, nausea, vomiting, and abdominal pain [8]. Although used as one of the characteristic features of pellagra, diarrhoea occurs in only half the cases of pellagra and is due to intestinal mucosal inflammation and atrophy [9]. Diarrhoea was not present in our patient´s case. Out of the traditional three Ds that typically characterise pellagra, only photosensitive dermatitis, and features of dementia were present in our patient. This presentation could have been due to a patchy involvement of the mucosal surface of the GI tract. A quick review of the literature revealed a paucity of data and a dearth of knowledge concerning the mechanism of constipation in pellagra, with only one paper, according to our knowledge reporting constipation in pellagra [10].

**Funding:***this work was supported through the Sub-Saharan African Network for TB/HIV Research Excellence (SANTHE), a DELTAS Africa Initiative [grant # DEL-15-006]. The DELTAS Africa Initiative is an independent funding scheme of the African Academy of Sciences (AAS)´s Alliance for Accelerating Excellence in Science in Africa (AESA) and supported by the New Partnership for Africa´s Development Planning and Coordinating Agency (NEPAD Agency) with funding from the Wellcome Trust [grant # 107752/Z/15/Z] and the UK government. The views expressed in this publication are those of the author(s) and not necessarily those of AAS, NEPAD Agency, Welcome Trust or the UK government*.

## Conclusion

INH preventive therapy in latent tuberculosis patients is commonly indicated in HIV patients who reside in high TB prevalence settings. A consideration of pellagra in patients on INH therapy whose diet is mostly maise-based will be beneficial, even if the classic 3 Ds of diarrhoea, dementia, and dermatitis are not wholly present. A timely diagnosis and prompt treatment of pellagra in this population can be lifesaving.
